# Prevalence of *Vibrio cholerae* in an Acute Watery Diarrhea Outbreak in Sulaymaniyah City, Iraq

**DOI:** 10.1155/ijm/5539834

**Published:** 2025-05-05

**Authors:** Hastyar Hamarashid Najmuldeen, Karzan Rafiq Sidiq, Fakher Karim Rahim, Karzan Taha Abubaker, Mazin Frya Faraj, Sima Rahman Qadir, Sina Khalil Ismael, Nozad Hussein Mahmood

**Affiliations:** ^1^College of Health Sciences, Cihan University-Sulaimaniya, Sulaymaniyah, Iraq; ^2^Department of Biology, College of science, University of Sulaimani, Sulaymaniyah, Iraq; ^3^Medical Laboratory Science Department, Charmo University, Sulaymaniyah, Iraq; ^4^Faculty of Internal Medicine, Osh State University, Osh, Kyrgyzstan; ^5^Laboratory Department, Shar Teaching Hospital, Sulaymaniyah, Iraq; ^6^Laboratory Department, Directorate of General Health of Sulaimaniya, Sulaymaniyah, Iraq; ^7^Research Center, Cihan University-Sulaimaniya, Sulaymaniyah, Kurdistan Region, Iraq

**Keywords:** 16s rRNA gene, cholera, epidemiology, genetic diversity, outbreak, *Vibrio cholerae*

## Abstract

Cholera is a life-threatening diarrheal disease caused by *Vibrio cholerae*, with recurring outbreaks in Iraq, including the Kurdistan Region. Despite its endemic nature, outbreaks have primarily been reported by the health sector without comprehensive molecular epidemiological investigations. Limited studies have characterized outbreak dynamics, prevalence, and antimicrobial resistance, hindering effective public health interventions. This study aimed to analyze the prevalence, molecular characteristics, and antibiotic resistance of *V. cholerae* isolates from the 2023 outbreak in Sulaymaniyah, Kurdistan, Iraq. A total of 1200 diarrheic stool samples were collected from Shar Hospital between July and October 2023. Bacterial isolation was performed using microbiological methods and automated VITEK 2 analysis, followed by serological identification (O1 and O139 antisera) and 16S rRNA gene sequencing. Antibiotic susceptibility testing (AST) was conducted to assess resistance patterns. The outbreak prevalence was 0.015%, with the highest infection rate in August (0.009%). The overall infection rate was 28.91% (347/1200), with the most affected age groups being 19–33 years (27.66%) and 34–48 years (26.22%). Infection was more common in females (55.6%) than males (44.4%). Phylogenetic analysis revealed high genetic similarity to the *V. cholerae* Kuwait1 strain, suggesting potential introduction from southern Iraq, possibly due to an influx of tourists. Furthermore, antibiotic susceptibility testing revealed that all *V. cholerae* isolates were susceptible to most tested antibiotics; however, complete resistance (100%) was observed against amikacin, amoxicillin, amoxiclav, nalidixic acid, and trimethoprim, with partial resistance (30%) to tetracycline. Cholera remains a major public health concern in Kurdistan, particularly in Sulaymaniyah, due to recurrent outbreaks. Molecular techniques provided crucial insights into outbreak tracking and genetic relatedness, while AST profiling highlighted the urgent need for revised treatment guidelines. Strengthening water sanitation, continuous antimicrobial resistance monitoring, and targeted public health interventions are essential for preventing future outbreaks.

## 1. Introduction

Cholera remains a significant public health concern worldwide, with an estimated 1.3 to 4 million cases and approximately 21,000 to 143,000 deaths annually [1–3]. The disease is caused by *Vibrio cholerae*, a Gram-negative bacterium that thrives in aquatic environments, particularly in coastal and estuarine water systems [4, 5]. Among its serogroups, *V. cholerae* O1 and O139 are responsible for epidemic cholera, producing cholera toxin (CT), which leads to acute secretory diarrhea. Transmission primarily occurs through contaminated food and water, making outbreaks common in regions with inadequate sanitation and limited access to clean water [6].

The current seventh cholera pandemic, caused by the *V. cholerae* O1 El Tor biotype, began in Indonesia in 1961 and continues to affect many regions, particularly in Asia and Africa [5–12].

In the year 2022, outbreaks of cholera were documented in several countries, including Pakistan, Cameroon, Tanzania, Malawi, Benin, Somalia, Afghanistan, Bangladesh, the Democratic Republic of Congo, Ethiopia, India, Nigeria, and Iraq [13–17]. In Iraq, recurrent cholera outbreaks have been reported from 1871 to the present, with the most recent outbreaks occurring in 2012, 2015, and 2022 [15, 16, 18–22]. The Kurdistan Region of Iraq has also faced multiple epidemics of acute watery diarrhea, including cholera, with Sulaymaniyah Governorate being the primary affected area during the latest outbreak in 2022 [23].

Despite ongoing surveillance and response efforts, knowledge gaps remain in understanding the epidemiology and molecular characteristics of *V. cholerae* strains in Iraq. Limited data exist regarding the genetic diversity of circulating strains, their phylogenetic relationships, and their resistance patterns. Addressing these gaps is crucial for effective outbreak management and control. Therefore, this study aimed to investigate the 2023 cholera outbreak in Sulaymaniyah City through isolation and identification of *V. cholerae* from suspected acute watery diarrhea cases using biochemical (*Vitek*) and serological methods. Additionally, molecular techniques were applied to confirm *V. cholerae* isolates and perform phylogenetic analysis to understand the genetic relatedness of the strains. Furthermore, an antibiotic sensitivity test was conducted to assess resistance patterns and determine the most effective treatment options for controlling the disease.

## 2. Methods

### 2.1. Location and Type of the Study

**Figure 1:**
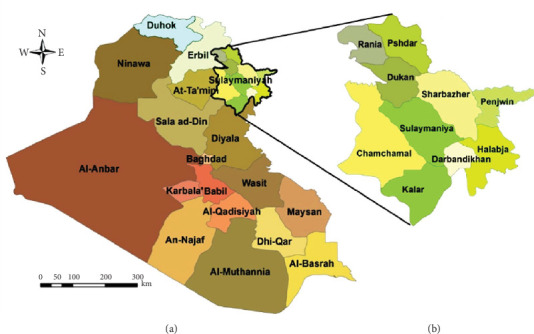
Map of Sulaymaniyah City (region of Kurdistan/Iraq) [24].

This descriptive case series study was carried out between July and October 2023 in Sulaymaniyah City, Kurdistan Region, the north of Iraq (Figure 1). Its geographical coordinates are 35°33⁣′42⁣^″^ north, 45°26⁣′27⁣^″^ east. Also, it is located at an elevation of 882 m above sea level with a total area of 1442 km^2^ [25].

### 2.2. Sample Collection

The World Health Organization (WHO) offices in the Kurdistan Region usually monitor the situation of acute watery diarrhea by collecting data via the health directorates in all the governorates. According to the WHO definition of cholera outbreak [6], a sudden rise in the daily cases of acute watery diarrhea, including rice watery diarrhea, makes the Ministry of Health practices the national cholera control plan (NCCP). The NCCP includes monitoring cholera, prevention measures, and stool sample processing.

The patients with acute watery diarrhea visited the primary healthcare centers (PHCC) and the hospitals, where the personal information and the stool samples were taken and transported in Cary–Blair medium to authorized laboratories in the nearest communicable disease prevention centers (CDPC) and hospitals across the city. The patient's demographic data such as gender, age, and address were recorded. Firstly, the data on the association between age and disease underwent an initial analysis using a primary clustering method (*k*-means). Through this method, nine age groups were established with the following ranges: 0–2, 3–5, 6–13, 14–18, 19–33, 34–48, 49–64, 65–78, and 79–98 years [26].

### 2.3. Bacterial Isolation and Identification

The samples were subjected to enrichment in alkaline peptone water and incubated at a temperature of 37°C for a minimum duration of 4 h in the hospital laboratory. The enhanced samples were subsequently introduced onto the selective thiosulfate-citrate-bile-salts-sucrose (TCBS) agar medium and placed in an incubator at a temperature of 40°C for a duration of 24 h. A solitary colony with a golden appearance was transferred to Kligler's iron agar (KIA) and placed in an incubator at a temperature of 37°C for a duration of 24 h. Simultaneously, the colony underwent an oxidase test. The colonies that tested positive for oxidase were then verified using serotyping techniques employing monovalent (O1 or O139) antisera. Furthermore, the VITEK 2 Compact System was utilized for additional confirmation.

### 2.4. Antibiotic Susceptibility Testing

The antimicrobial susceptibility of the bacterial isolates was assessed using the Kirby–Bauer disk diffusion method, as described by Bauer et al. Overnight, bacterial cultures grown in nutrient broth were standardized to a 0.5 McFarland turbidity level (equivalent to 1.5 × 10^8^ CFU/mL) using a McFarland densitometer (Grant-Bio) [27]. Subsequently, 100 *μ*L of each adjusted bacterial suspension was evenly spread onto Mueller–Hinton agar plates using a sterile cotton swab, followed by a short drying period.

According to the WHO standard guide, the twenty antibiotic disks were applied: amikacin 30 *μ*g, amoxicillin 25 *μ*g, amoxiclav 30 *μ*g, azithromycin 15 *μ*g, aztreonam 30 *μ*g, ceftazidime 30 *μ*g, ceftriaxone 10 *μ*g, nalidixic acid 30 *μ*g, tobramycin 10 *μ*g, tetracycline 30 *μ*g, cefotaxime 30 *μ*g, cephalexin 30 *μ*g, chloramphenicol 30 *μ*g, ciprofloxacin 5 *μ*g, doxycycline 30 *μ*g, rifampicin 5 *μ*g, ciprofloxacin 5 *μ*g, levofloxacin 5 *μ*g, norfloxacin 5 *μ*g, piperacillin 100 *μ*g, and trimethoprim 5 *μ*g (Bioanalyse & HIMEDIA, India). The disks were placed onto the agar surface using sterile forceps. The inoculated plates were then incubated at 37°C for 18 h [28]. After incubation, the diameters of the inhibition zones around the antibiotic disks were measured and interpreted based on the Clinical and Laboratory Standards Institute (CLSI) guidelines [29].

### 2.5. Colony PCR (Polymerase Chain Reaction)

Colony PCR was performed on 35 randomly selected samples, previously identified as *V. cholerae*, using a published method [30]. The 16S rRNA gene was targeted to confirm the identification of the clinical isolates. In a concise overview, 500 *μ*L of an overnight culture underwent centrifugation at 10,000 × *g* for 3 min. The resulting pellet was then resuspended in 300 *μ*L of nuclease-free water, followed by heating at 95°C for 10 min and a subsequent centrifugation at 10,000 × *g* for 2 min.

For the PCR reaction mixture, 10 *μ*L of master mix (Trans Gene Biotech, China), 0.2 *μ*L each of the forward primer (27 Fp, AGAGTTTGATCCTGGCTCAG) and reverse primer (534 Rp, ATTACCGCGGCTGCTGG) from Macrogen company (South Korea), 4 *μ*L of crude DNA, and the remaining volume adjusted to 20 *μ*L using nuclease-free water were combined. The PCR program (Applied Biosystems, United States) initiated with an initial denaturation step at 94°C for 5 min, followed by 30 cycles of denaturation at 94°C for 30 s, primer annealing at 55°C for 30 s, and primer extension at 72°C for 30 s. The program concluded with a final extension step at 72°C for 7 min.

After PCR amplification, the products were visualized through 1% (w/v) agarose gel electrophoresis, and the DNA concentration was determined using Thermo Scientific's NANODROP 2000 spectrophotometer. For sequencing of the 16S rRNA gene of *V. cholerae*, a premix was prepared by mixing 2 *μ*L of either forward or reverse primers (10 pmol *μ*L^−1^) with 15 *μ*L of the purified PCR product (20 ng *μ*L^−1^). The premixes were sent to DNA sequencing service provider (Macrogen Company, South Korea). The sequencing data were analyzed by the NCBI-BLAST server (https://blast.ncbi.nlm.nih.gov/Blast.cgi).

### 2.6. Phylogenetic Analysis

Genetic variation at the 16S rRNA locus was explored to determine the evolutionary genetic relationships among *Vibrio cholerae* isolates. The observed variants were compared with reference sequences deposited in the NCBI-BLAST server (https://blast.ncbi.nlm.nih.gov/Blast.cgi). Subsequently, BLAST results of the observed variants were aligned with gap corrections in Clustal Omega–based tools [31]. A phylogenetic tree was generated using the neighbour-joining process in MEGA 11 software [32].

## 3. Results

### 3.1. Identification of *Vibrio cholerae*

**Figure 2:**
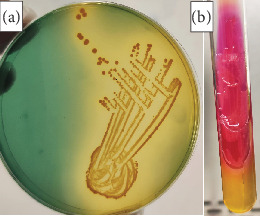
Bacteriological identification of *Vibrio cholerae*. (a) Well-isolated golden-shining colonies of *V. cholerae* on TCBS medium. (b) Kligler's iron agar slant, showing an acid/alkaline reaction with no gas production and no hydrogen sulfide (H_2_S) formation.

The isolated pathogen *V. cholerae* formed well-defined golden-shining colonies on TCBS agar (Figure 2a). Further biochemical characterization confirmed the presence of *V. cholerae* through a positive oxidase reaction and sugar fermentation, indicated by changes in acid/alkaline balance, with no hydrogen sulfide (H_2_S) or gas production in KIA medium (Figure 2b). Additionally, identification using the VITEK 2 compact system and serological analysis of 347 isolates (out of 1200 clinical samples) confirmed that the current outbreak was caused by *V. cholerae* O1 serogroup, El Tor biotype, Ogawa serotype.

### 3.2. Prevalence of Cholera

**Figure 3:**
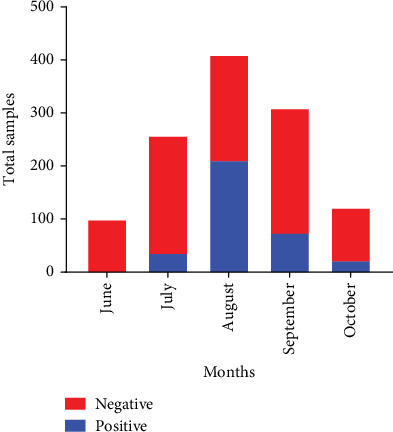
Frequency distribution of cholera-positive and negative cases at Shar Hospital from June to October 2023.

During the outbreak, 1200 suspected cases of acute watery diarrhea were admitted to Shar Hospital in Sulaymaniyah City, Iraq. Among this cohort, 347 samples tested positive for the cholera bacterium, with the infection rate of 28.91% among the suspected cases and the prevalence of 0.015%. The outbreak was first documented in July, with 14.34% (37 out of 258 cases) testing positive. The infection rate then increased significantly in August, peaking at 51.7% (212 out of 410 cases). In September and October, the rates declined to 24.19% (75 out of 310 cases) and 18.85% (23 out of 122 cases), respectively (Figure 3).

**Figure 4:**
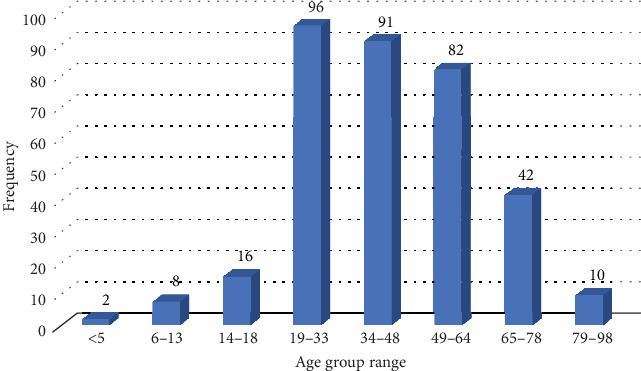
Frequency distribution of cholera cases across different age groups.

The average age of cholera patients was 42.7 ± 18.28 years. The highest number of cases (27.66%, 96 out of 347) occurred in individuals aged 19–33 years, followed by those aged 34–48 years (26.22%, 91 out of 347) and 49–64 years (23.63%, 82 out of 347). The incidence decreased in older age groups, with 12.10% (42 out of 347) in individuals aged 65–78 years and 2.88% (10 out of 347) in those aged 79–98 years. The lowest incidence was recorded in 0.58% (2 out of 347) of young children in those under 5 years and 2.31% (8 out of 347) in children aged 6–13 years (Figure 4).

**Figure 5:**
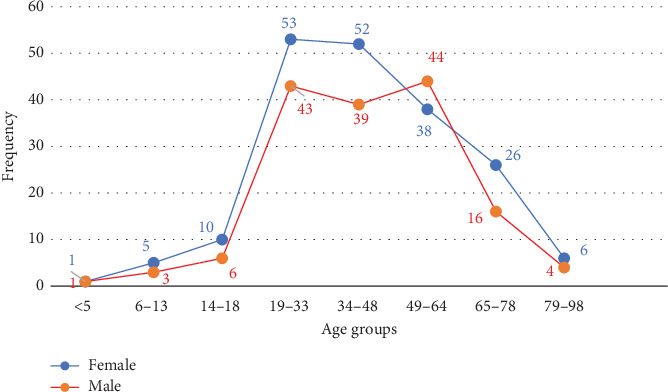
Frequency distribution of cholera cases among males and females across different age groups.

Regarding gender distribution, 55.6% (193 out of 347) of cases occurred in females, compared to 44.4% (145 out of 347) in males, indicating a higher prevalence in females (Figure 5).

Molecular identification based on 16S rRNA gene sequencing confirmed the accurate identification of biochemically and serologically characterized *Vibrio cholerae* strains. Sequence analysis showed 99% similarity to *V. cholerae* strain Kuwait1 (JF731344.1) in the NCBI-BLAST database. The sequences were deposited in GenBank (NCBI) under accession numbers PV162850, PV162851, PV162852, PV162862, and PV162863. Multiple sequence alignment of the partial 16S rRNA gene sequences from local isolates revealed high genetic similarity to globally reported strains.

**Figure 6:**
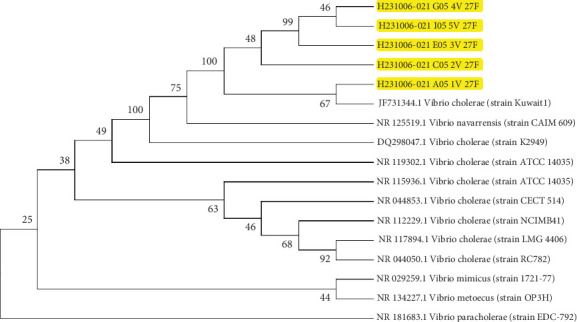
Phylogenetic tree analysis of *V. cholerae* isolates based on 16S rRNA gene sequences using MEGA 11.0.

Phylogenetic analysis further demonstrated a strong genetic relationship between local *V. cholerae* isolates and Kuwait1 (JF731344.1) strains (Figure 6). The isolates clustered within a single clade, with a common node at 100, suggesting high genetic conservation. Notably, isolate A051V 27F shared a common ancestor with *V. cholerae* strain Kuwait1, indicating a potential link between local and regional strains.

### 3.3. Antibiotic Susceptibility Testing

The antimicrobial susceptibility testing of *V. cholerae* clinical isolates revealed varying degrees of resistance and sensitivity against different antibiotic classes. All tested isolates demonstrated sensitivity to the third-generation cephalosporins, including ceftriaxone, ceftazidime, and cefotaxime, as well as other *β*-lactam antibiotics such as cephalexin and piperacillin. Additionally, the isolates exhibited susceptibility to aztreonam, a monobactam antibiotic, which further underscores the effectiveness of certain *β*-lactam agents against *V. cholerae*.

Among the aminoglycosides, a contrasting pattern was observed, with the isolates being resistant to amikacin but sensitive to tobramycin. In the fluoroquinolone category, ciprofloxacin, levofloxacin, and norfloxacin were all effective, showing complete susceptibility. Macrolide susceptibility testing revealed that azithromycin remained an effective treatment option for *V. cholerae*.

Chloramphenicol and doxycycline also showed high efficacy, as all isolates were sensitive to these antibiotics. However, 30% of the tested isolates exhibited resistance to tetracycline, suggesting the potential emergence of resistance mechanisms affecting this antibiotic class. Rifampicin, an RNA polymerase inhibitor, exhibited complete effectiveness, whereas nalidixic acid demonstrated resistance. Furthermore, resistance was recorded against amoxicillin and amoxicillin/clavulanic acid, suggesting potential *β*-lactamase-mediated resistance mechanisms within these isolates.

## 4. Discussion

Cholera continues to be one of the most widespread infections, characterized by the sudden onset of watery diarrhea. In many developing countries, the high disease occurrence is primarily attributed to poor hygiene and sanitation practices, along with delayed treatment and prevention due to inadequate healthcare infrastructure [33, 34]. The prevalence of cholera in Iraq has increased as a consequence of the first and second Gulf wars, leading to its endemic status since 1991.

In this outbreak, the phenotypic characterization of the isolated *V. cholerae* showed golden-shining colonies on TCBS medium, a positive oxidase reaction, sucrose fermentation, and the absence of H_2_S and gas production on KIA. These characteristics align with the standard traits of *V. cholerae*, as previously reported [35]. The isolation of the *V. cholerae* O1 serogroup, El Tor biotype, and Ogawa serotype in this outbreak suggests that the Ogawa serotype may become endemic, as the 2022 outbreak was also caused by the same serotype [25]. The Ogawa serotype is a predominant cause of cholera in several regions worldwide [35, 36]. The findings confirm that *V. cholerae* is a major pathogenic agent responsible for acute watery diarrhea in urban areas, the prevalence of 0.015%, and an infection rate of 28.91% among hospitalized patients. These incidence rates are comparable to those in neighbouring regions with similar environmental and cultural factors. A previous study revealed that cholera epidemics in Iraq recur approximately every 4–5 years, with notable outbreaks occurring in 2003, 2007, 2012, and 2015 [11]. The 2015 cholera outbreak in Iraq resulted in 1500 deaths across 18 provinces [33]. In 2022, Sulaymaniyah Province experienced a cholera outbreak in June and July, leading to the admission of 4754 suspected cases to Shar Hospital [35].

The seasonal increase in cholera cases during summer is linked to water shortages and contamination, as rising temperatures, increased water demand, and reliance on unregulated water sources collectively contribute to the spread of waterborne diseases. Studies have shown that the direct discharge of untreated sewage and industrial waste into water bodies leads to microbial contamination, particularly during peak summer months [34]. Water bodies pose a significant risk of fecal contamination and cholera transmission [37]. The recurring summer cholera epidemic in Sulaymaniyah may be linked to prolonged water storage in inadequately cleaned or uncovered tanks [38, 39], as well as the frequent use of contaminated swimming pools [40], particularly in warm summer temperatures, which promote bacterial growth and spread.

The substantial increase in cases in August (51.7%), followed by a gradual decline in the following months, may reflect the impact of prevention measures and seasonal effects. This trend aligns with previous studies highlighting the role of environmental factors, such as temperature and rainfall, in cholera incidence [7, 41, 42]. Similarly, in August 2022, Iraq reported 783 cholera cases and four deaths, with Kirkuk, Baghdad-Rasafa, and Thi Qar recording the highest case numbers at 450, 193, and 52, respectively [14], indicating the spread of infection to other regions, including Sulaymaniyah, possibly facilitated by the influx of tourists from cholera-endemic areas in central and southern Iraq [17, 43].

The 2023 outbreak affected both genders, aligning with findings from Sabir et al. [35]. The highest infection rate was observed among adults aged 19–48 years, a pattern consistent with other cholera-endemic regions [17, 35, 44].

In contrast, the lower incidence of cholera in children under 5 years in our study, compared to findings by Deen et al. and Ali et al., may be influenced by differences in study settings, population demographics, and sample collection criteria [34, 44]. Improved maternal immunity and breastfeeding practices could also provide passive protection, reducing susceptibility in infants. Additionally, immunity levels, exposure patterns, and hygiene practices may further shape the demographic distribution of cases [45].

Molecular analysis demonstrated that local isolates shared 99% genetic identity with global strains such as *V. cholerae* strain Kuwait1 (JF731344.1). This suggests a potential link between local and regional strains, possibly originating from southern Iraq. The potential link between local and regional strains could be attributed to similar climatic conditions and movement of populations between Iraq and Kuwait. Mukhopadhyay et al. studied the *Vibrio cholerae* O1 genotype of ctxB and suggested that this strain was imported from Iraq to Kuwait [46]. Multiple sequence alignment further confirmed the genetic similarity among *V. cholerae* isolates, with slight variations attributed to horizontal gene transfer [47]. These findings support previous research emphasizing the genetic stability of *V. cholerae* over time [48].

Antimicrobial susceptibility testing showed that third-generation cephalosporins (ceftriaxone, ceftazidime, cefotaxime), *β*-lactam antibiotics (cephalexin, piperacillin), and fluoroquinolones (ciprofloxacin, levofloxacin, norfloxacin) remain highly effective against *V. cholerae* [49, 50]. However, resistance to amoxicillin and amoxicillin/clavulanic acid suggests the presence of *β*-lactamase-producing strains [51, 52]. The emergence of tetracycline resistance is concerning, though doxycycline remains effective [53].

Resistance to trimethoprim, an antifolate antibiotic that inhibits dihydrofolate reductase, indicates possible mutations or horizontal gene transfer affecting folate metabolism pathways [51, 52]. Additionally, variability in aminoglycoside response, such as resistance to amikacin but sensitivity to tobramycin, highlights the need for precise antibiotic selection [52, 54]. Resistance mechanisms, including enzyme-mediated modifications, efflux pumps, and mutations affecting membrane permeability, may reduce intracellular aminoglycoside and antifolate antibiotic concentrations, contributing to differential susceptibility patterns [51, 52, 54].

Environmental factors significantly influence antibiotic resistance patterns in *V. cholerae*. The presence of antibiotic residues in wastewater and aquatic ecosystems has been associated with the acquisition of resistance genes [55–57]. Studies detecting antibiotic-resistant *V. cholerae* strains in surface water highlight environmental contamination as a reservoir for resistance determinants [58]. Key resistance mechanisms involve mobile genetic elements, such as integrative conjugative elements (ICEs) and plasmids, which facilitate the horizontal transfer of resistance genes [53].

It can be concluded that the prevalence of cholera in Sulaymaniyah remains low (0.015%), with seasonal outbreaks peaking in warmer months. The 2023 outbreak, caused by *V. cholerae* O1 Ogawa, closely relates to a Kuwaiti strain, suggesting transmission via a tourist. Genetic analysis highlights the need for continuous surveillance to track outbreak sources and transmission dynamics.

Rising antimicrobial resistance, particularly to tetracyclines, underscores the urgency of antibiotic stewardship and resistance monitoring. To prevent future outbreaks, strengthening water sanitation, early preventive measures before June, and targeted public health interventions are crucial.

## Data Availability

Data availability is provided if requested.
